# Effects of low temperature on flowering and the expression of related genes in *Loropetalum chinense* var. *rubrum*

**DOI:** 10.3389/fpls.2022.1000160

**Published:** 2022-11-15

**Authors:** Damao Zhang, Qianru Chen, Xia Zhang, Ling Lin, Ming Cai, Wenqi Cai, Yang Liu, Lili Xiang, Ming Sun, Xiaoying Yu, Yanlin Li

**Affiliations:** ^1^ Hunan Agricultural University, College of Horticulture, Changsha, Hunan, China; ^2^ Engineering Research Center for Horticultural Crop Germplasm Creation and New Variety Breeding, Ministry of Education, Changsha, China; ^3^ Hunan Mid-Subtropical Quality Plant Breeding and Utilization Engineering Technology Research Center, Changsha, China; ^4^ School of Economics, Hunan Agricultural University, Changsha, China; ^5^ Beijing Key Laboratory of Ornamental Plants Germplasm Innovation and Molecular Breeding, National Engineering Research Center for Floriculture, Beijing Laboratory of Urban and Rural Ecological Environment, Key Laboratory of Genetics and Breeding in Forest Trees and Ornamental Plants of Ministry of Education, School of Landscape Architecture, Beijing Forestry University, Beijing, China; ^6^ Kunpeng Institute of Modern Agriculture, Foshan, China

**Keywords:** low temperature, flowering, bud, gibberellin, FLC, FT, Ap1

## Abstract

**Introduction:**

*Loropetalum chinense* var. *rubrum* blooms 2-3 times a year, among which the autumn flowering period has great potential for exploitation, but the number of flowers in the autumn flowering period is much smaller than that in the spring flowering period.

**Methods:**

Using ‘Hei Zhenzhu’ and ‘Xiangnong Xiangyun’ as experimental materials, the winter growth environment of *L. chinense* var. *rubrum* in Changsha, Hunan Province was simulated by setting a low temperature of 6-10°C in an artificial climate chamber to investigate the effect of winter low temperature on the flowering traits and related gene expression of *L. chinense* var. *rubrum*.

**Results:**

The results showed that after 45 days of low temperature culture and a subsequent period of 25°C greenhouse culture, flower buds and flowers started to appear on days 24 and 33 of 25°C greenhouse culture for ‘Hei Zhenzhu’, and flower buds and flowers started to appear on days 21 and 33 of 25°C greenhouse culture for ‘Xiangnong Xiangyun’. The absolute growth rate of buds showed a ‘Up-Down’ pattern during the 7-28 days of low temperature culture; the chlorophyll fluorescence decay rate (Rfd) of both materials showed a ‘Down-Up-Down’ pattern during this period. The non-photochemical quenching coefficient (NPQ) showed the same trend as Rfd, and the photochemical quenching coefficient (QP) fluctuated above and below 0.05. The expression of *AP1* and *FT* similar genes of *L. chinense* var. *rubrum* gradually increased after the beginning of low temperature culture, reaching the highest expression on day 14 and day 28, respectively, and the expression of both in the experimental group was higher than that in the control group. The expressions of *FLC*, *SVP* and *TFL1* similar genes all decreased gradually with low temperature culture, among which the expressions of *FLC* similar genes and *TFL1* similar genes in the experimental group were extremely significantly lower than those in the control group; in the experimental group, the expressions of *GA3* similar genes were all extremely significantly higher than those in the control group, and the expressions all increased with the increase of low temperature culture time.

**Discussion:**

We found that the high expression of gibberellin genes may play an important role in the process of low temperature promotion of *L. chinense* var. *rubrum* flowering, and in the future, it may be possible to regulate *L. chinense* var. *rubrum* flowering by simply spraying exogenous gibberellin instead of the promotion effect of low temperature.

## 1. Introduction

*Loropetalum chinense* var. *rubrum*, an evergreen shrub or small tree (Xia et al., 2020), which with bright leaves and as an important landscape application tree species in Hunan and even the middle and lower reaches of the Yangtze River (Runmin and Xiaoying, 2012). The flower color of *L.chinense* var. *rubrum* is absolutely gorgeous (Zhang et al., 2022)and annual flowering 2-3 times. The spring flowers is in March-April, while autumn flowers are in October-November. Although the large amount of flowers opened in spring, the flowering period of *L.chinense* coincides with other ornamental plants, which resulting in no competitive advantage in the application direction of flowering. The autumn flowering period coincides with the National Day, and its bright red flower color complements the atmosphere of the National Day celebration, which provides a new idea for the development of its flowering value. However, the number of spring flowers is much larger than that of autumn flowers in the natural environment (Bao et al., 2007). Whether this phenomenon is related to the low temperature in winter has not yet been proved. Clarifying this relationship has certain help for the excavation of the flower value of *L.chinense* var. *rubrum.*

Numerous studies have shown that photoperiod and low temperature induction are the main inducers of flowering in most plants, and that changes in these environmental conditions ultimately regulate flowering through substances in the leaves called ‘florigen’ (Taoka et al., 2013). Low temperature in winter is an important environmental condition for inducing and accelerating the flowering of many plants (Lina et al., 2003; Zhonghua et al., 2006). Plants start the transformation from vegetative growth to reproductive growth under the induction of low temperature, which is also called vernalization (Alexandre and Hennig, 2008). *FLC* plays an important role in floral transition by encoding a MADS-box transcription factor (Changsheng et al., 2021). It was shown that the expression of the *FLC*-like gene *VRN2* was suppressed in wheat leaves after vernalization induction, reducing the suppression of a MADS box protein-containing *VRN1* gene (with high homology to the downstream flowering regulator *AP1* in Arabidopsis) in leaves, thereby promoting flowering (Yan et al., 2004). The results showed that the transcription level of *FLC* gene was higher in cabbage without vernalization. At this time, cabbage showed late flowering traits. After 10 days of induction at 4°C, the expression level of *FLC* gene in cabbage with early flowering traits decreased significantly (Kim et al., 2007; Kitamoto et al., 2013). During vernalization, *FLC* not only suppresses the transcriptional expression of *FT* by binding to the promoter of the downstream gene *FT*, but also hinders the regulation of the floral meristem gene *AP1* by *FT* and inhibits the flower-forming transition, but also binds to the transcription factor *SVP* to form heterodimer, which jointly negatively regulates the expression of *FT* gene, thereby inhibiting flowering (Mateos et al., 2015; Luo et al., 2019; Kinmonth-Schultz et al., 2021). *FT* gene can also inhibit the expression of *FLC* gene in vegetative and reproductive growth stages, especially in seed development stages (Chen and Penfield, 2018). In 2007, it was shown that FT proteins in leaves are ‘florigen’ that bind to FD proteins in stem tips and promote the expression of genes in downstream floral meristems, thus promoting flowering (Taoka et al., 2011). *FT* and *TFL1* belong to the same family of PEBPs and share 98% amino acid sequence similarity, but they have opposing functions (Kobayashi et al., 1999; Karlgren et al., 2011; Sriboon et al., 2020). The balance between *FT* and *TFL1* determines the early and late flowering of pear trees, and they are antagonistic. The flowering time is controlled by the combination of competition and *FD* to activate the transcription and expression of downstream floral meristem gene *AP1* (Abe et al., 2005; Hanano and Goto, 2011; Ito et al., 2022). AP1 gene, as an important member of the ABCDE model of floral development, which can identify and accept a large number of signal pathways. These signals can guide floral meristem development into flowers by inhibiting or promoting the expression of *AP1* (Wellmer and Riechmann, 2010; O’Maoileidigh et al., 2014).

The initiation of flower bud differentiation marks the transition of plants from vegetative growth to reproductive growth. In this process, a large number of nutrients, endogenous hormones and other contents are changed (Blázquez et al., 1998; Han et al., 2016; Rizza and Jones, 2019). The changes in gibberellin content are interspersed throughout the developmental stages of flowers and make a difference (Shihao et al., 2008; Wang et al., 2015). When exogenous gibberellin is applied, it induces flowering in long-day plants instead of low temperature, while when endogenous *GA* synthesis is blocked, flowering is delayed (Qiaoxia et al., 2019). This is the well-known gibberellin pathway that promotes flowering by inhibiting *SVP* gene expression and positively regulating *FT* and *SOC1* genes (Mateos et al., 2015). At the same time, the increase of gibberellin content in the process of flowering induction can improve the photosynthetic capacity of plant leaves (Weicai et al., 2014), then providing more energy for the flowering process and ensuring the successful completion of the flowering process (Christiaens et al., 2015).

This experiment was conducted with a new varieties of *L.chinense* and a *L.chinense* var. *rubrum*, and the experiment was carried out after the end of their autumn flowering period. The low-temperature environmental conditions in winter were simulated by low-temperature incubation at 6-10°C, and the effect of winter low temperature on the flowering of red frond was initially investigated by combining parameters such as the number of flowers, chlorophyll fluorescence parameters, and the expression of related genes.

## 2. Materials and methods

### 2.1 Materials

The experimental materials included a new variety ‘Xiangnong Xiangyun’ selected by Hunan Agricultural University (variety registration number: 20201102, variety registration agency: State Forestry and Grassland Administration) and ‘Hei Zhenzhu’. Among them, ‘Xiangnong Xiangyun’ is the *L. chinense* of the *Loropetalum*, which blooms 1-2 times a year, with a large number of flowers, and the petal color is pure white or light beige; ‘Hei Zhenzhu’ is the *L.chinense* var. *rubrum* of the *Loropetalum*, which blooms 1-2 times a year, blooms in large numbers, and the petal color is magenta or rose. All experimental materials were the progeny of cuttings from both cultured for two years with stable genetic background. The experimental sites include the artificial climate room at Hunan Agricultural University (Room 007, 11th Teaching Building, Hunan Agricultural University) and the intelligent greenhouse in the flower base of Hunan Agricultural University.

The experiment started in November 2020 after the end of the autumn flowering period of Loropetalum Chinense.Eight pots each of ‘Xiangnong Xiangyun’ and ‘Hei Zhenzhu’ with good growth and consistent crown size were selected and numbered X.T1-X.T8 and H.T1-H.T8, respectively. After 5 days of gradient low temperature exercise (Starting from 25°C, adjust the parameters of the artificial climate chamber to reduce the ambient temperature by 3°C per day and end with 10°C), the above 16 pots of material were sequentially transferred to the artificial climate chamber for low temperature culture. The artificial climate chamber was set for a total of five cycles, 8:00-12:00, 8°C, light; 12:00-14:00, 10°C, light; 14:00-18:00, 8°C, light; 18:00-8:00 the next day, 6°C, dark. At the same time, the ambient temperature in the smart greenhouse was maintained at 25°C. After 42 days of continuous incubation in the artificial climate chamber, the plants were moved into the smart greenhouse sequentially after 3 days of gradient warming in the artificial climate chamber and incubated in the same environment as the control plants for subsequent observation (Starting from 10°C, adjust the parameters of the artificial climate chamber to increase the ambient temperature by 5°C per day and end with a warming of 25°C).During the experimental cycle, the water and fertilizer management was kept consistent for all plants, watering once every 3 days and fertilizing moderately once every 15 days.

### 2.2 Methods

#### 2.2.1 Observation of buds

After the experiment started, the maximum transverse diameter and the longest longitudinal diameter of their shoots were measured sequentially every 7 days using a digital vernier caliper, and the shoots from the same part of the same plant were taken each time and repeated three times and recorded. After the low-temperature culture ended and the room-temperature culture started, the status of each plant bud was observed once every 3 days, and the number and time of present buds and flowers were recorded.

#### 2.2.2 Determination of chlorophyll fluorescence parameters

Chlorophyll fluorescence parameters were measured randomly every 3 days after the start of the experiment using a hand-held chlorophyll fluorometer on leaves from the same location of the same plant. For the measurement, the leaves to be measured are first held in a dark adaptation using a leaf clamp for 30 minutes. After dark adaptation, chlorophyll fluorescence parameters were measured sequentially using the ‘OJIP’ and ‘NPQ3’ programs in the instrument, and each measurement was repeated three times.

#### 2.2.3 Detection of gene expression

During the experiment, 7-8 pieces of apical leaves from different plants of the same species were taken every 7 days in lyophilization tubes, snap-frozen in liquid nitrogen and stored in an ultra-low temperature refrigerator at -80°C. Two tubes were sampled and stored each time. Refer to the StarSpin HiPure Plant RNA Mini Kit (GeneStar, Beijing) kit instructions to extract total RNA. The cDNA was synthesized by referring to the Evo M-MLV (Ekore, Hunan) reverse transcription kit instructions. And we compared the protein sequences of the related genes of *L.chinense* var. *rubrum* in NCBI (https://www.ncbi.nlm.nih.gov/Structure/bwrpsb/bwrpsb.cgi), and preliminarily identified the similar functions of the related genes and similar genes through the protein domain, and the identification results were provided in the form of supplementary figures, primers were designed using Beacon Designer 8 (**Table 1**).The PCR reaction system was 2X SYBR Green Pro Taq HS Premix*5µL, 0.8µL each of upstream and downstream primers (10µmol/L), 1µL of cDNA, and ddH2O to make up to 10µL.PCR amplification conditions were: step 1, 95°C for 30 seconds, step 2, 95°C for 5 seconds, 60°C for 30 seconds, 72°C for 10 minutes for 40 cycles, 65°C for 5 seconds, 95°C for 5 seconds, 3 repetitions, and the relative expression of the target gene was calculated according to the 2^-ΔCt^ method.

**Table 1:** Real-time fluorescence quantitative PCR primer information for *L.chinense* var. *rubrum*.

Gene type	Gene		Primer sequence (5´-3´)
Internal reference genes	β-actin2	F	CCACAAGGCTTATTGATAGAAT
		R	CAATGGTTGAACCTGAATACT
*AP1*-like gene	augustus40012	F	CCAGCTTGATAATGCTCTTAA
		R	GTGCCTTCTCCTTCTCTT
*FT*-like genes	augustus63326	F	ATCTTAGGACCTTCTACACTC
		R	AATATCAGTCACCAACCAATG
	augustus34660	F	GTCTCTACCGATCTCTACAC
		R	CTTCTGGAATGTCAACAACA
*SVP*-like gene	augustus15211	F	CAGCCATCTCCATCTCTT
		R	ACACGACTCAATCCAACT
*FLC*-like gene	augustus27517	F	GTTTCTTCCTCAAATTCAACTTT
		R	CTACCATCCTCATTATTCTTCTTAT
*TFL1*-like gene	augustus62587	F	CTAGAAGAGAGGTGGTGAC
		R	CAGTGAAGAAGGTGGAGTA
*GA*-like genes	augustus49213	F	CACATTCTCGGCATTATCAA
		R	TCACCACCTTGTCTTCTC
	augustus58706	F	TCCTACCTCCTTAACTATACTTC
		R	CCTGTTCACTATTGCTCTATG
	augustus63711	F	GGTGAGCACTGTGGATAT
		R	CCTCGCAATAGTCCTGAT

### 2.3 Statistical analysis

The experimental data were statistically analyzed using SPAS 22.0, the Duncan method was used for multiple comparisons, Origin 2019b was used for graph drawing.

## 3 Results

### 3.1 Effect of low temperature on flowering

Combined with **Tables 2** and **3**, ‘Xiangnong Xiangyun’ and ‘Hei Zhenzhu’, which were cultured at low temperature and then at room temperature, were able to flower, and both had the maximum number of flower buds of 33 and 22 on days 27 and 30, respectively, after being cultured at room temperature. The maximum number of flowering was 39 and 16 on day 36, respectively. The flowering process was the same for both, but ‘Xiangnong Xiangyun’ had more buds and flowers than ‘Hei Zhenzhu’. In contrast, the two experimental materials, which were continuously cultured at 25°C, did not show buds or flowers throughout the experimental cycle.

**Table 2:** Number of flower buds and time to bud emergence under normothermic (25°C) culture after low-temperature induction.

Date	Time/day	Number of flowering bud	
		Hei Zhenzhu	Xiangnong Xiangyun
20210108	20	0	0
20210109	21	0	6
20210112	24	5	13
20210115	27	21	33
20210118	30	22	32
20210121	33	14	25
20210124	36	4	3
20210125	37	0	0

No flower buds were seen for the two experimental materials that were continuously incubated at 25°C throughout the experimental cycle.

**Table 3:** Flowering number and flowering time under normothermic (25°C) culture after low-temperature induction.

Date	Time/day	Number of flowering bud	
		Hei Zhenzhu	Xiangnong Xiangyun
20210117	29	0	0
20210118	30	0	1
20210121	33	8	19
20210124	36	16	39
20210127	39	6	12
20210130	42	0	2
20210131	43	0	0

The two experimental materials incubated continuously at 25°C throughout the experimental cycle did not see flowering.

### 3.2 Effect of low temperature on bud growth rate and photosystem

**Figure 1:**
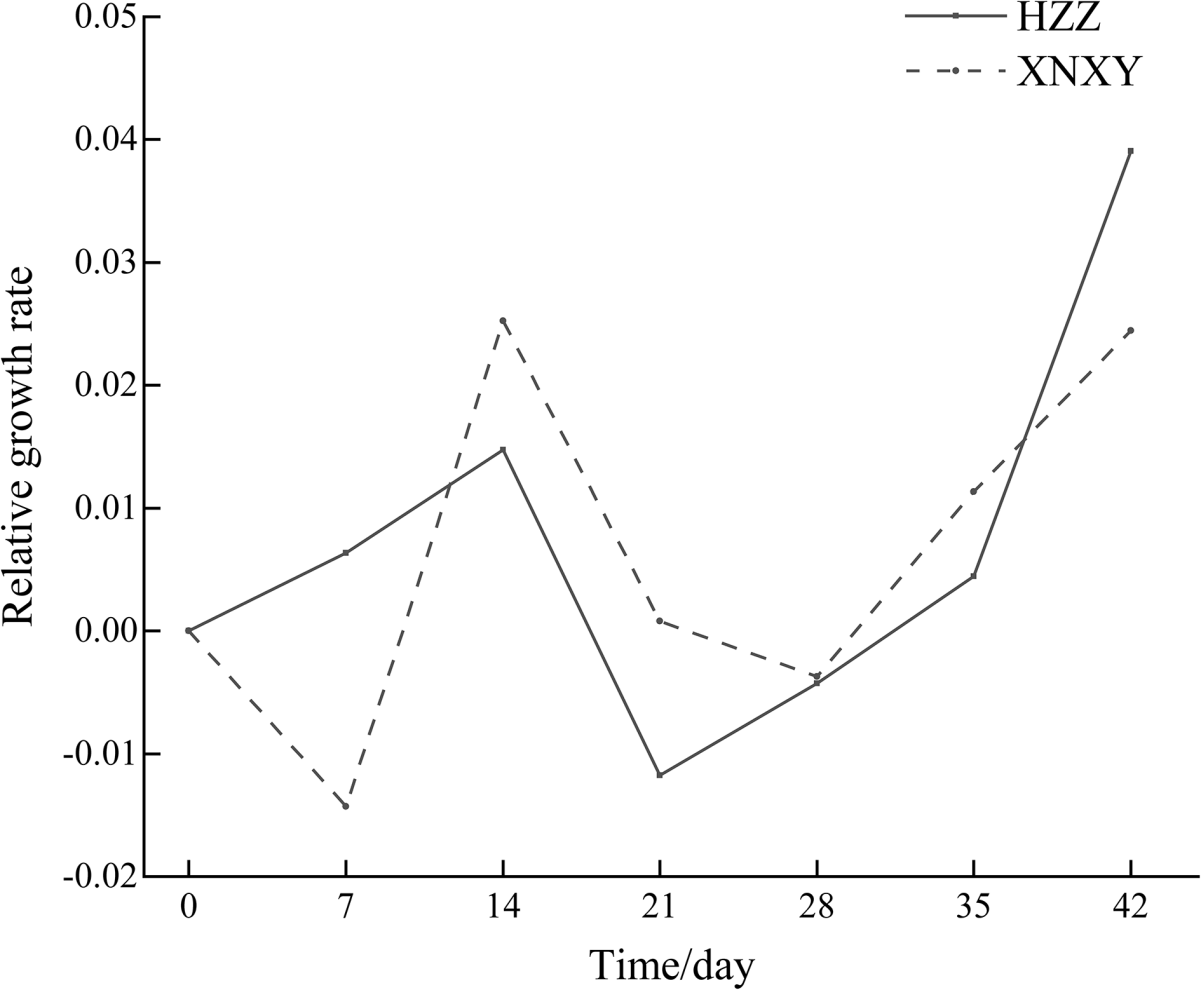
Absolute growth rate of shoots of ‘Xiangnong Xiangyun’ and ‘Hei Zhenzhu’ during low temperature culture. ‘XNXY’ stands for ‘Xiangnong Xiangyun’, ‘HZZ’ stands for ‘Hei Zhenzhu’.

**Figure 2:**
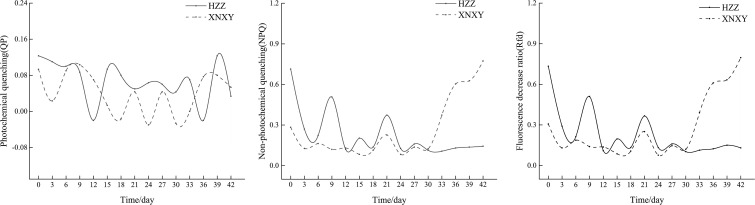
Changes in chlorophyll fluorescence parameters of 'Xiangnong Xiangyun' and 'Hei Zhenzhu' during low temperature culture. ‘XNXY’ stands for ‘Xiangnong Xiangyun’, ‘HZZ’ stands for ‘Hei Zhenzhu’.

From **Figure 1**, the absolute growth rates of ‘Xiangnong Xiangyun’ and ‘Hei Zhenzhu’ showed a ‘Up-Down’ pattern during the 7-28 days of low temperature culture, after which the absolute growth rates of shoots continued to increase. In combination with the Rfd curve of chlorophyll fluorescence decay rate (right 1) and the NPQ curve of non-photochemical quenching coefficient (middle) in **Figure 2**, the Rfd curves of ‘Xiangnong Xiangyun’ and ‘Hei Zhenzhu’ showed an overall pattern of ‘Down-Up- Down’. And reached a higher level on day 21 of this period, the trend of NPQ curve was consistent with Rfd, and the value of photochemical quenching coefficient QP fluctuated regularly above and below 0.05.

### 3.3 Effect of low temperature on the expression of flowering-related genes

#### 3.3.1 Effect of low temperature on *AP1* gene expression

**Figure 3:**
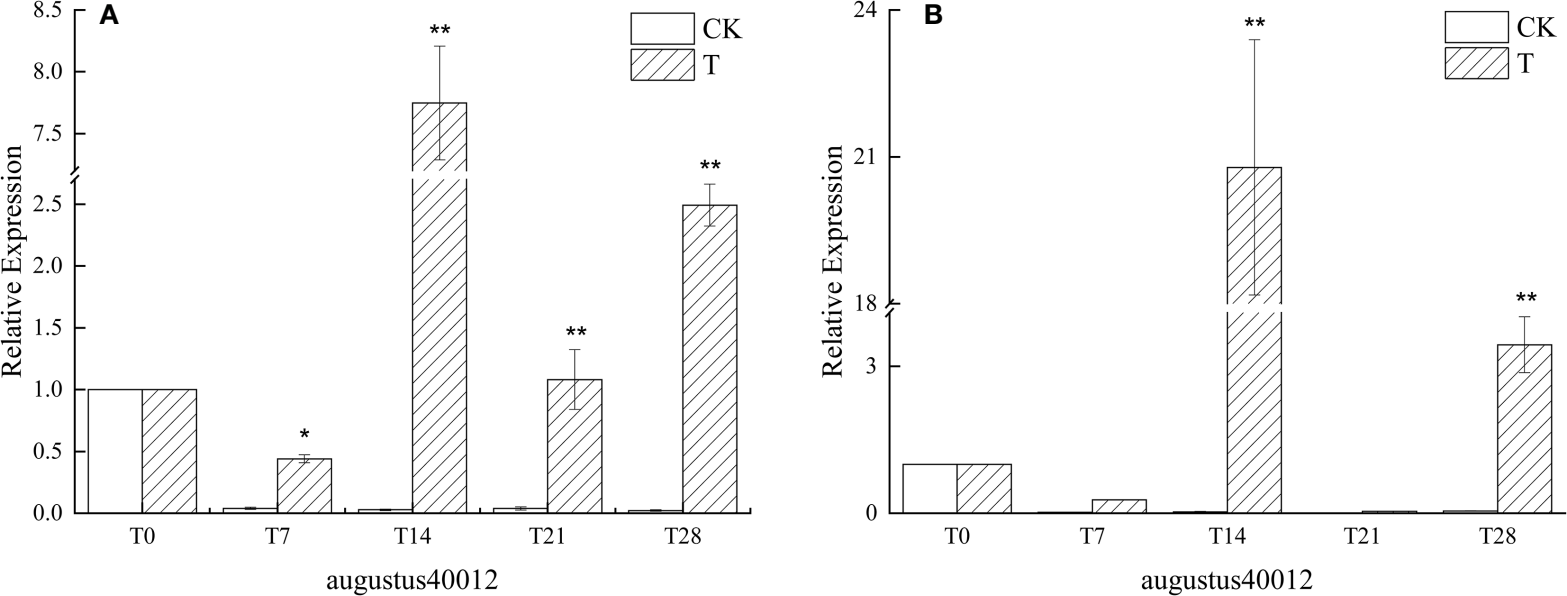
Relative expression of the similar gene augustus40012 of *AP1* in the leaves of plants with different treatments in *L. chinense* var. *rubrum.* ‘**(A)**’ represents the relative expression of similar genes of *AP1* in leaves of ‘Xiangnong Xiangyun’ under different treatments. ‘**(B)**’ represents the relative expression of similar genes of *AP1* in leaves of ‘Hei Zhenzhu’ under different treatments. ‘T’ represents the low-temperature treatment group and ‘CK’ represents the blank control group treated at 25°C. '*' significant difference, '**' extremely significant difference.

The expression of agustus40012, a similar gene of *AP1* in *L. chinense* var. *rubrum*, was generally in an up-regulated state as the low-temperature induction time progressed. In particular, the expression reached the highest level in the leaves of ‘Xiangnong Xiangyun’ and ‘Hei Zhenzhu’ on day 14 after the beginning of low-temperature induction, the expression in the leaves of ‘Xiangnong Xiangyun’ is 7.74 times that of the T0 period (**Figure 3A**), and the expression in the leaves of ‘Hei Zhenzhu’ is 20.78 times that of the T0 period (**Figure 3B**), and the expression in the leaves of both reaches the highest state at this time. The expression at T28 was still 2.39 and 3.44 times higher than that at T0, which was still in an up-regulated expression state. Moreover, in the period from T7 to T28, the expression of this gene in the T group was significantly higher than that in the CK group. It can also be seen that in the CK group, the expression of AP1-like genes in the leaves of both experimental subjects in the T0 period is higher than that in the T7 to T28 period.

#### 3.3.2 Effect of low temperature on *FT* gene expression

**Figure 4:**
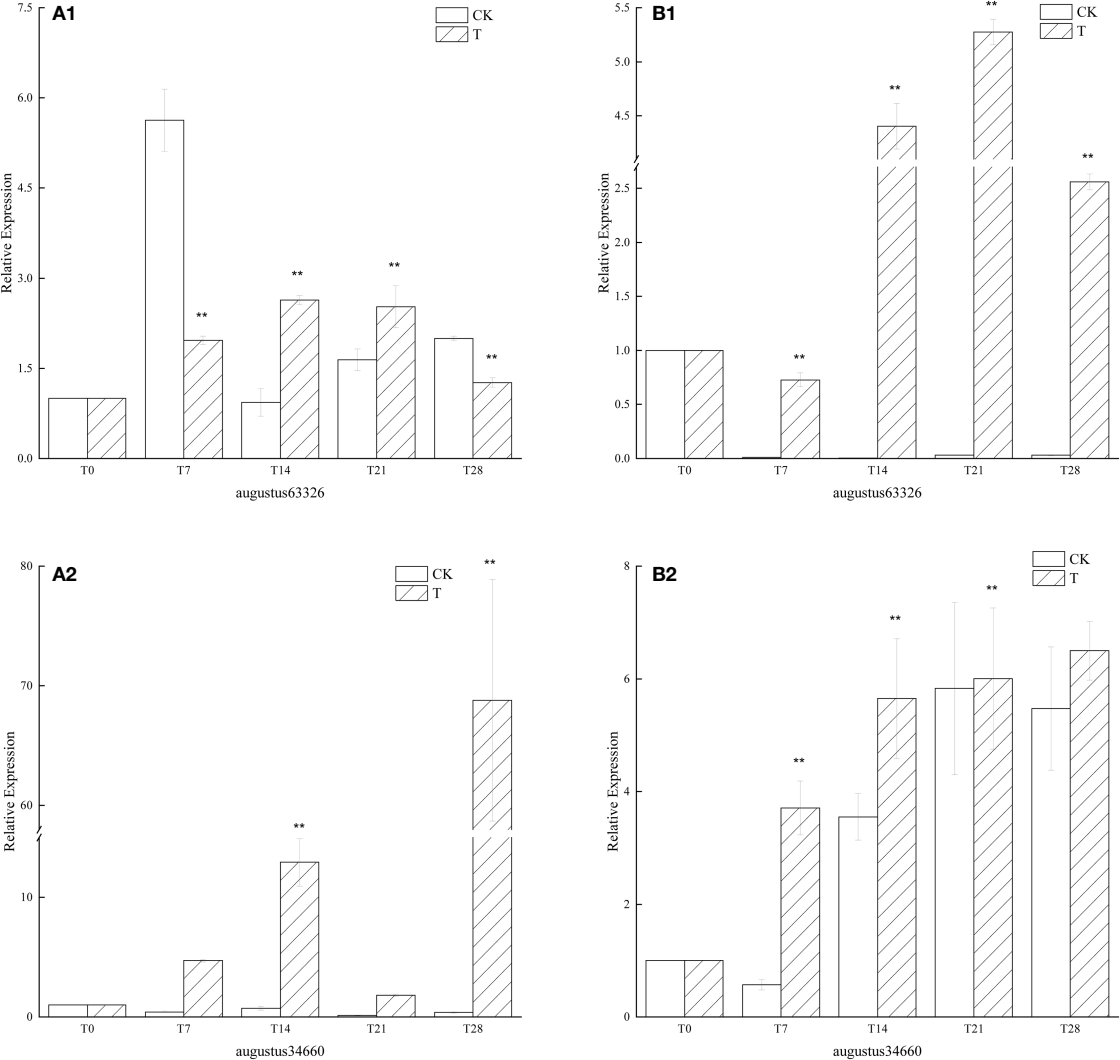
Relative expression of similar genes augustus63326 and augustus34660 of *FT* in the leaves of plants with different treatments in *L. chinense* var. *rubrum.* ‘**(A)**’ represents the relative expression of similar genes of *FT* in leaves of ‘Xiangnong Xiangyun’ under different treatments. ‘**(B)**’ represents the relative expression of similar genes of *FT* in leaves of ‘Hei Zhenzhu’ under different treatments. ‘T’ represents the low-temperature treatment group and ‘CK’ represents the blank control group treated at 25°C. ** extremely significant difference.

Since the beginning of low temperature culture, *FT* similar genes in the low temperature treatment group had similar expression phenomena in ‘Xiangnong Xiangyun’ and ‘Hei Zhenzhu’. Both genes showed up-regulated expression in general, but the expression of augustus63326 appeared to increase and then decrease during the low-temperature culture. In ‘Xiangnong Xiangyun’, the expression of augustus63326 was 2.63 times higher at T14 than at T0 and 2.52 times higher at T21 than at T0, and the gene had higher expression in both periods (**Figure 4A1**). In ‘Hei Zhenzhu’, augustus63326 is expressed 4.40 times more in the T14 period than in the T0 period, and augustus63326 is expressed in the T21 period is 5.28 times that in the T0 period, both of which also have higher expression (**Figure 4B1**). The expression of augustus63326 in both subjects decreased in the T28 period, but it was still 1.26 times and 2.56 times that of the T0 period. The expression of this gene in the T28 period was 68.79 times (**Figure 4A2**) and 6.50 times (**Figure 4B2**) higher than that in the T0 period, both reaching the highest values in ‘Xiangnong Xiangyun’ and ‘Hei Zhenzhu’. At the same time, the expression of augustus34660 was significantly higher than that of the CK group in all periods under low temperature treatment. It can be found that except for augustus63326 of ‘Xiangnong Xiangyun’, the expression of FT-like genes in the low-temperature treatment group was higher than that in the CK group in all periods.

#### 3.3.3 Effect of low temperature on *SVP*, *FLC* and *TFL1* gene expression

**Figure 5:**
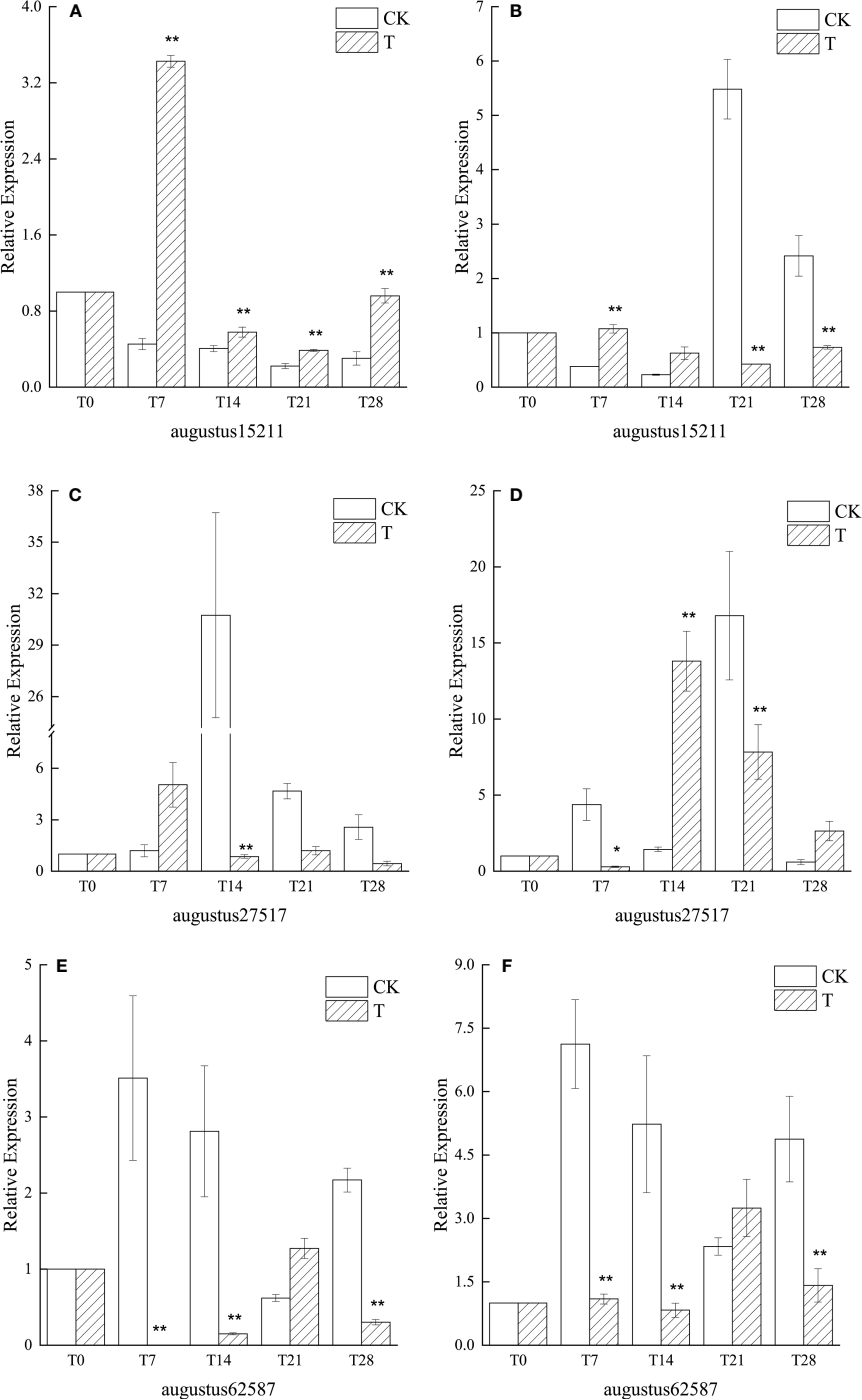
Relative expression of similar genes augustus15211, augustus27517, and augustus62587 of *SVP*, *FLC*, and *TFL1* in leaves of plants with different treatments of *L. chinense* var. *rubrum.* ‘**(A)**’, ‘**(C)**’ and ‘**(E)**’ represent the relative expressions of *SVP*, *FLC* and *TFL1* similar genes in leaves of ‘Xiangnong Xiangyun’ under different treatments, respectively. ‘**(B)**’, ‘**(D)**’ and ‘**(F)**’ represent the relative expression of similar genes of *SVP*, *FLC* and *TFL1* of ‘Hei Zhenzhu’ in leaves under different treatments, respectively. ‘T’ represents the low-temperature treatment group and ‘CK’ represents the blank control group treated at 25°C. '*' significant difference, '**' extremely significant difference.

After the experiment started, with the experimental time, the similar genes of *SVP* and *FLC* in the low temperature treatment group were consistently down-regulated in the leaves of ‘Xiangnong Xiangyun’ and ‘Hei Zhenzhu’. The expression of augustus15211 reached the lowest expression at T21, 0.39 (**Figure 5**) and 0.42 (**Figure 5**) times that of T0, respectively. augustus27517 had lower expression at T28, 0.44 (**Figure 5**) and 2.64 (**Figure 5**) times that of T0, respectively, and The expression of augustus27517 was overall highly significant higher in the CK-treated group than in the low-temperature-treated group. Except for the T21 period, the expression of augustus62587, a similar gene of *TFL1*, was extremely significantly lower in both ‘Xiangnong Xiangyun’ and ‘Hei Zhenzhu’ under low temperature treatment than in the CK treatment group. In the T21 period, the expression of augustus62587 showed extreme values in both materials, at which time the highest expression of augustus62587 appeared in both materials under low temperature treatment, which were 1.27 (**Figure 5**) and 3.25(**Figure 5**) times higher than in the T0 period, respectively. While the lowest expression of augustus62587 appeared in both materials in the CK group, which were 0.62 (**Figure 5**) and 2.33(**Figure 5**) times higher than that in the T0 period, respectively.

#### 3.3.4 Effect of low temperature on *GA* gene expression

**Figure 6:**
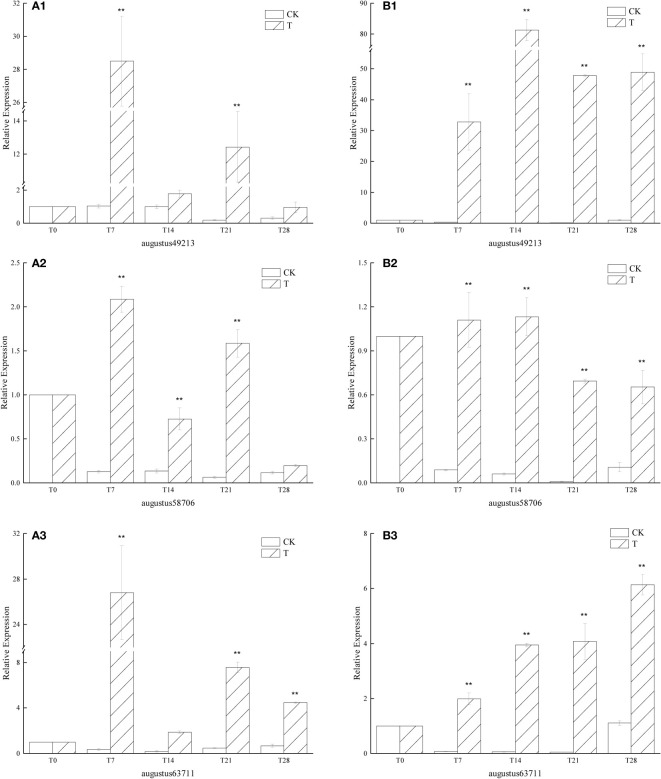
Relative expression of similar genes augustus49213, augustus58706 and augustus63711 of *GA* in the leaves of plants with different treatments of *L. chinense* var. *rubrum.* ‘**(A)**’ represents the relative expression of *GA* similar genes in leaves of ‘Xiangnong Xiangyun’ under different treatments. ‘**(B)**’ represents the relative expression of *GA* similar genes in leaves of ‘Hei Zhenzhu’ under different treatments. ‘T’ represents the low-temperature treatment group, ‘CK’represents the blank control group treated at 25°C. ** extremely significant difference.

Overall, the expression of the similar genes of *GA* in the low-temperature treatment group increased sharply within 7 days after the start of the low-temperature treatment. Among the three, except for augustus58706, the expression of the other two genes under low temperature treatment was very different from that in the period before low temperature treatment was started, and the extreme values were: the expression in the T7 period was 28.50 times that of the T0 period (**Figure 6A1**), the expression in the T14 period was 81.31 times that in the T0 period (**Figure 6B1**), and the expression in the T21 period was 26.81 times that in the T0 period (**Figure 6A3**), The expression in the T28 period is 6.13 times that in the T0 period (**Figure 6B3**). The expression of augustus58706 under low temperature treatment was not much different from that in the non-low temperature treatment period, and the highest expression was 2.09 times (**Figure 6A2**) and 1.13 times (**Figure 6B2**) of the T0 period, respectively. In general, the expression of the T group was significantly higher than that of the CK group in all periods. It can also be seen that the expression of all three in the CK group is at a very low level.

## 4 Discussion

On the one hand, some deciduous and evergreen plants escape the cold by losing their leaves and changing their leaf structure to minimize respiration and photosynthesis during winter (Oquist and Huner, 2003), and some plants, such as hostas (Hawryzki et al., 2011), escape the cold by cutting themselves above ground and keeping the lower part of the ground in a dormant state (Dogramaci et al., 2010; Yu et al., 2010). On the other hand, through long-term adaptation, most plants have evolved a trait that needs to be induced by low winter temperatures to flower and bear fruit, a phenomenon called vernalization.

In this study, it was found that after low-temperature induction, the *L. chinense* var. *rubrum* can still flower successfully even if the autumn flowering period has passed, while no flowering was seen in the same batch of *L. chinense* var. *rubrum* cultured at a constant temperature of 25°C. It shows that appropriate low-temperature induction can promote not only flowering and fruiting of some plants, but also flowering of *L. chinense* var. *rubrum*. Similarly, when Satsuma mandarins are grown continuously at 25°C, they have only nutritional growth, whereas when they are cultivated at a relatively low temperature of 15°C there is a distinction between nutritional and reproductive growth (Agusti et al., 2022). Combined with the absolute growth rate of buds in **Figure 1**, we found that when the low temperature started, the growth rate of buds was very low, and the plant responded to the low temperature environment by weakening its various physiological activities at low temperature (Sønstebya and Heideb, 2019), indicating that the various physiological activities of *L. chinense* var. *rubrum* itself had been weakened at this time. Subsequently, with the continuation of low temperature culture time, the growth rate of buds began to rise, at this time the *L. chinense* var. *rubrum* has adapted to the low temperature environment, and by the low temperature induced after the promotion of buds began to undergo transformation, in the low temperature buds from the original dormant state or nutrient growth state to reproductive growth state. After that the growth rate of the buds of the *L. chinense* var. *rubrum* decreased and then increased again. During this stage, the buds may pass through a period of low temperature induction before first completing part of the process of transition to reproductive growth and then entering dormancy again. After a period of time, with the continuation of the low temperature induction, the buds are prompted to complete the subsequent stage of floral bud differentiation.

The inhibition of plant growth and physiological functions in low temperature environments is related to the reduction in the activity of photosystem II and photosystem I at low temperatures (Tang et al., 2020). The energy conversion efficiency of photosystem II can be visualized by chlorophyll fluorescence parameters (Maxwell and Johnson, 2000), in agriculture, real-time monitoring of chlorophyll fluorescence parameters of crops enables accurate measurement of crop photosynthesis, allowing more accurate prediction of agricultural productivity and climate effects on crop yield. And the efficiency of photosystem II can usually be reflected by the value of Fv/Fm, while Rfd is a more sensitive indicator of plant vigor and photosynthetic rate than Fv/Fm (Lysenko et al., 2014; Shin et al., 2017). It can be seen that the Rfd values of the *L. chinense* var. *rubrum* in this experiment decreased sharply after the beginning of the experiment, indicating that the photosynthetic efficiency and physiological activities of the *L. chinense* var. *rubrum* at the initial stage of low temperature decreased rapidly, which is consistent with the result that the growth rate of the buds of the *L. chinense* var. *rubrum* decreased at the beginning of the experiment. From the 6th day of the experiment, the trend of Rfd was opposite to the growth rate of the shoots, and the growth rate of the shoots started to decrease when the value of Rfd increased. When plants suddenly enter reproductive growth, nutritional growth will suffer inhibition, inhibited nutritional growth will in turn inhibit reproductive growth (Gaaliche et al., 2011; Li and Zhang, 2012; Rosati et al., 2018), reproductive growth intervenes, the increase in photosystem activity of the leaves to obtain more nutrients to supply reproductive growth (Yang et al., 2012a; Yang et al., 2019). At this time, the supply of nutritional growth of buds is reduced, and the growth rate is reduced, followed by the inhibition of reproductive growth by nutritional growth, and the combined effect of the low temperature environment causes a decrease in photosynthetic efficiency and a decrease in the value of Rfd. This was repeated until the flower bud differentiation was completed in the low temperature environment. In the low temperature environment, the trend of NPQ is consistent with Rfd (Shin et al., 2021), therefore, the NPQ curve of *L. chinense* var. *rubrum* under low temperature treatment has a similar trend to the Rfd curve. The magnitude of the QP value reflects the photosynthetic efficiency of photosystem II to a certain extent (Shouren, 1999; Xin and Jirui, 2012), and the QP value of *L. chinense* var. *rubrum* under low-temperature environment remained above and below 0.05, indicating that photosystem II of redbud suffered a stronger inhibition under low-temperature conditions. Combined with **Figure 2** , the phenomenon that ‘Xiangnong Xiangyun’ has more buds and flowers can be explained because the Rfd value of ‘Xiangnong Xiangyun’ showed a rapid increasing trend in the late low temperature culture, indicating that its photosystem II recovered some activity at this time. The photosynthetic efficiency of ‘Xiangnong Xiangyun’ was much higher than that of ‘Hei Zhenzhu’, which caused ‘Xiangnong Xiangyun’ to obtain more organic assimilated material to provide more energy for reproductive growth. Eventually, ‘Xiangnong Xiangyun’ had more flower buds to complete the transformation.

Among the four pathways that regulate flowering in plants, the main signal for the vernalization pathway is low temperature from the environment (Bond et al., 2011). The most important gene in the vernalization pathway is *FLC*, which encodes a MADS-box transcription factor and a flowering repressor, and the higher the expression, the stronger the repression of flowering (Yang et al., 2012b), its expression is inhibited by low temperatures during vernalization (Helliwell et al., 2006). *FLC* can inhibit flowering by interacting with *SVP* to form a dimer (Mateos et al., 2015), and by binding to the first intron region of *FT*, it strongly inhibits *FT* transcription and thus prevents bud differentiation (Li et al., 2008). *FT* is highly conserved in flowering plants and it can integrate regulatory signals from different pathways to regulate flowering (Sheng et al., 2013; Song et al., 2013), it promotes flowering when its expression is upregulated and loses its ability to promote flowering when it is downregulated (Nishikawa et al., 2007). As for *TFL1*, which belongs to the same PEPB family, its function is contrary to that of *FT* due to the alteration of a key amino acid residue in its PEPB structural domain (Klintenäs et al., 2012), and the two regulate the expression of downstream flowering genes such as *AP1* by competitively binding FD proteins (Corbesier et al., 2007; Zhu et al., 2020). As can be seen from **Figure 5**, the similar genes augustus27517 and augustus15211 of *FLC* and *SVP*, both of which showed a decreasing trend in expression since the beginning of low-temperature culture, while in CK both of which were maintained at high levels, indicating that the low-temperature culture environment in the experiment suppressed the expression of similar genes of *FLC* and *SVP* in *L. chinense* var. *rubrum*. The similar gene augustus62587 of *TFL1* was also at a low expression level under low temperature conditions, and was at a disadvantage when competing with *FT* to bind FD protein and therefore promoted flowering, while augustus62587 in the CK group was at a high expression level and was at an advantage in the competition and therefore inhibited flowering. Combined with **Figure 4**, the expression of both *FT* similar genes in the low temperature treatment group was consistently elevated, while the expression of *FT* genes in the CK group were maintained at low levels. the high level of *FT* expression again indicated that the low temperature treatment suppressed the expression of *FLC* and *SVP*, while the high level of *FT* expression was at an advantage relative to *TFL1* when competing for FD proteins, thus promoting bud differentiation and flowering.

During plant flower formation, various regulatory signals are eventually fed back to *AP1* through different pathways (Quan et al., 2019). The above *FT* integrator signal is also ultimately delivered to the *AP1* gene *via* the FD protein in order to function (Wigge et al., 2005). *AP1* is a floral meristem characteristic gene in the ABCDE model of flower development and belongs to the MADS-box family, which plays an extremely important role in the flower-forming transition (Li et al., 2021). As shown in **Figure 3** , the similar gene augustus40012 of *AP1* maintained a higher expression level in the low temperature treatment group, while the expression level of augustus40012 was at a lower expression level in the CK group, indicating that the above genes together increased the expression level of augustus40012 under low temperature through different pathways and ways, thus promoting flowering.

Flower bud differentiation is the process of transition from nutritional to reproductive growth, and the process of breaking the hormonal balance in the plant (Munne-Bosch and Lalueza, 2007; Pan et al., 2012). Among the many endogenous plant hormones, gibberellin (GA) has been shown to be closely related to flowering (Zhang et al., 2018). In the gibberellin flowering pathway, exogenous gibberellin regulates the up-regulated expression of AP1 gene to promote flowering in Arabidopsis under short sunlight with LFY protein as an intermediate (Eriksson et al., 2006). It has been shown that the internal active gibberellin content of *Brassica juncea* gradually increased to a peak during its floral bud differentiation after vernalization treatment, indicating that low temperature also promotes the expression of GA genes (Shang et al., 2017). Combined with **Figure 6**, in this study, the similar genes augustus49213, augustus58706 and augustus63711 of *GA* were maintained at higher expression levels during the low temperature treatment, while the gibberellin content in the CK group in all periods was always lower than that in the T0 period, indicating that low temperature promoted the expression of augustus49213, augustus58706 and augustus63711 in *L. chinense* var. *rubrum.* The up-regulation of gibberellin-related gene expression and low temperature together activated the gibberellin flowering pathway and promoted flowering in *L. chinense* var. *rubrum.*

## 5 Conclusion

**Figure 7:**
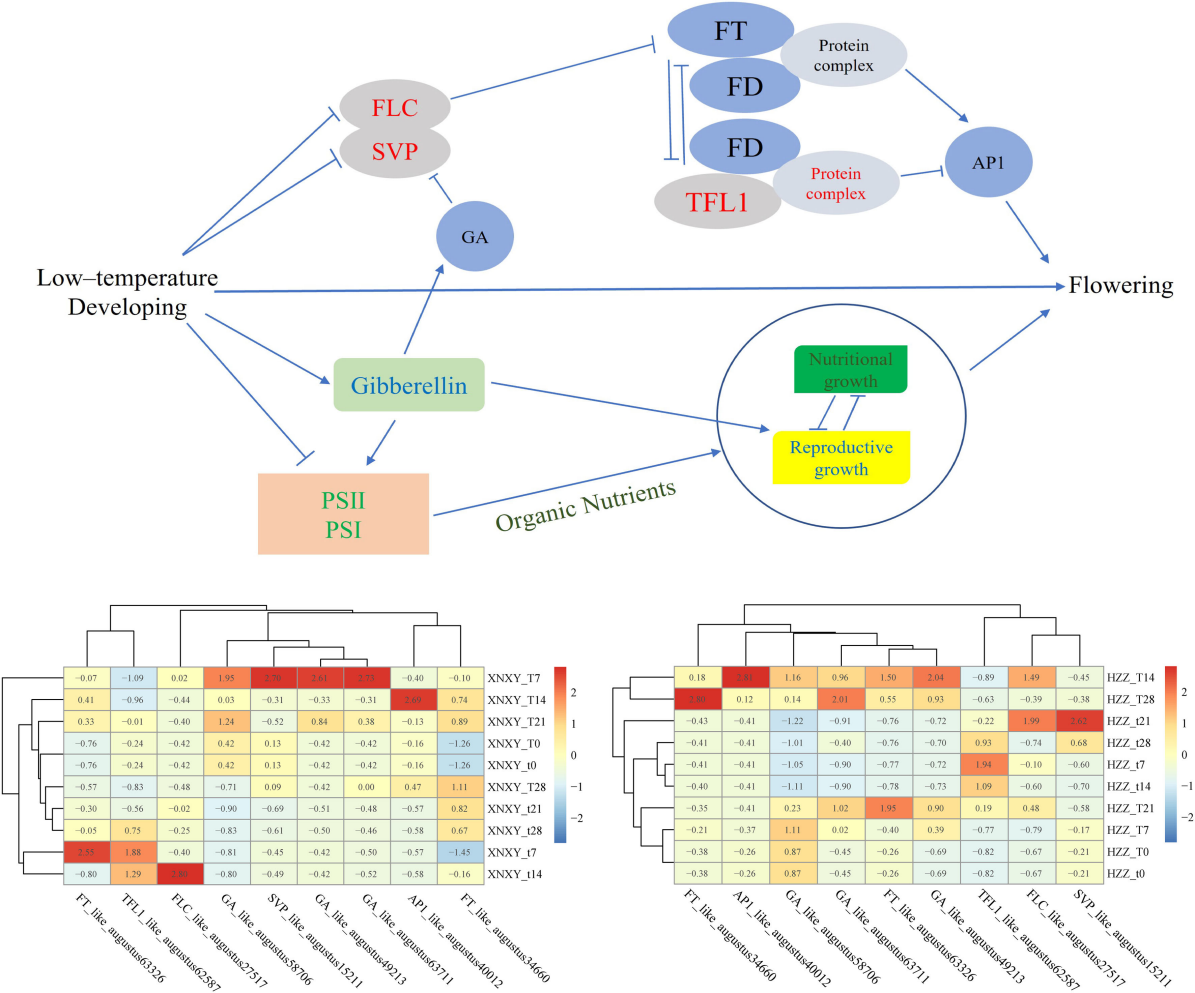
Regulation model for low temperature promotion of flowering in Eryngium. The gene expression values in the heat map are obtained from the original values after homogenization by the scale function. ‘T’ represents the low-temperature treatment group and ‘t’ represents the blank control group treated at 25°C, ‘XNXY’ represents the ‘Xiangnong Xiangyun’ and ‘HZZ’ represents the ‘Hei Zhenzhu’.

In summary, low-temperature culture is a reliable way to promote the flowering of *L. chinense* var. *rubrum* as a form of regulation, and low-temperature induction in winter is one of the reasons why the number of flowers in the spring flowering period of *L. chinense* var. *rubrum* is more than the number of flowers in the autumn flowering period. The molecular regulatory network of low temperature-promoted flowering is complex (Bond et al., 2011), the most critical of which is that the genes *FLC* and *SVP*, which are repressors of flowering formation, suffer from repression of transcript levels at low temperatures (Yan et al., 2006), and that low temperature conditions promote the expression of *GA* genes activating the gibberellin flowering pathway, which together activate the expression of the downstream flowering-promoting gene FT, which is capable of integrating multiple signals (Xu et al., 2012), and ultimately the *FT* gene positively regulates the expression of the floral meristem gene *AP1* to promote flowering transformation (Wellmer and Riechmann, 2010), based on these results we can obtain **Figure 7**. Through our research, we can lay the foundation for the subsequent development and use of exogenous gibberellin spraying to regulate the flowering of *L. chinense* var. *rubrum*, so that the goal of making full use of autumn flowers of *L. chinense* var. *rubrum* can be realized.

## Data availability statement

The original contributions presented in the study are included in the article/**Supplementary Material**. Further inquiries can be directed to the corresponding authors.

## Author contributions

DZ conceived and designed the experiments, performed the experiments, analyzed the data, prepared figures and/or tables, authored or reviewed drafts of the paper, and approved the final draft. QC performed the experiments, analyzed the data, authored or reviewed drafts of the paper, and approved the final draft. XZ and LL analyzed the data, authored or reviewed drafts of the paper, and approved the final draft. MC and LX conceived and designed the experiments, authored or reviewed drafts of the paper, and approved the final draft. WC analyzed the data, prepared figures and/or tables, and approved the final draft. YL performed the experiments, prepared figures and/or tables, and approved the final draft. MS, XY and YLL conceived and designed the experiments, prepared figures and/or tables, authored or reviewed drafts of the paper, and approved the final draft.

## Funding

The work is funded by the Innovation training program for college students of Hunan Agricultural University (XCX2021044), National Innovation and Entrepreneurship Training Program for College Students (202112653017X), Open Project of Horticulture Discipline of Hunan Agricultural University (2021YYXK001), The Forestry Science and Technology Innovation Foundation of Hunan Province for Distinguished Young Scholarship (XLKJ202205), Innovation and Entrepreneurship Training Program of Hunan Province for College Students (201941937227), The Found of Changsha Municipal Science and Technology Bureau (KQ2202227), Hunan Provincial Natural Science Youth Foundation Project (2020JJ5264), Hunan Provincial Education Department Teaching reform Project (2021JGYB101), Hunan Agricultural University Teaching reform research project (XJJG-2020-071).

## Conflict of interest

The authors declare that the research was conducted in the absence of any commercial or financial relationships that could be construed as a potential conflict of interest.

## Publisher’s note

All claims expressed in this article are solely those of the authors and do not necessarily represent those of their affiliated organizations, or those of the publisher, the editors and the reviewers. Any product that may be evaluated in this article, or claim that may be made by its manufacturer, is not guaranteed or endorsed by the publisher.
