# Supplementary figures and images for: Effects of low temperature on flowering and the expression of related genes in Loropetalum chinense var. rubrum
Source: Front Plant Sci. 2022 Nov 15;13:1000160. doi: 10.3389/fpls.2022.1000160 (PMC9705732; doi:10.3389/fpls.2022.1000160)

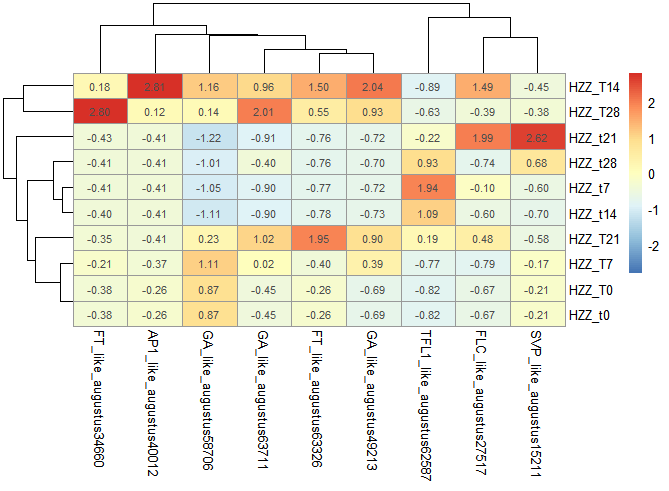

Supplement: Supplementary file 2 [file DataSheet_2.zip › pheatmap_data_and_code/3f84856c8f288771ee028033bce36a5.png]

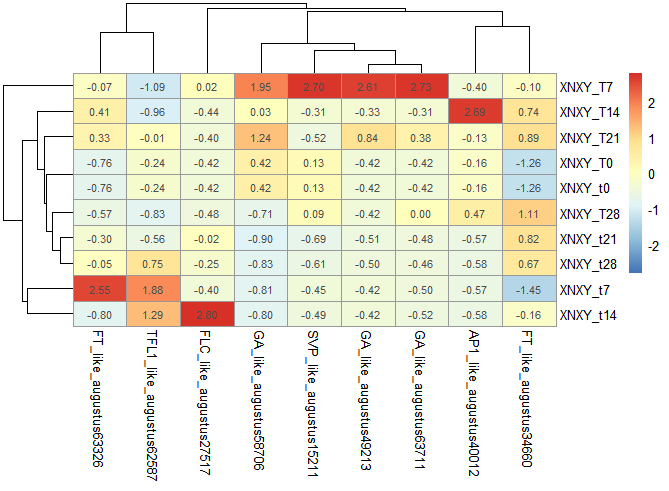

Supplement: Supplementary file 2 [file DataSheet_2.zip › pheatmap_data_and_code/cb7ea54c00a39c7493b9022e4a49ccd.png]
