# Supplementary material for: Effects of low temperature on flowering and the expression of related genes in Loropetalum chinense var. rubrum
Source: Front Plant Sci. 2022 Nov 15;13:1000160. doi: 10.3389/fpls.2022.1000160 (PMC9705732; doi:10.3389/fpls.2022.1000160)

augustus40012.t1-AP1

**Protein classification:** K-box domain-containing protein may have a coiled-coil structure, and a possible role in multimer formation

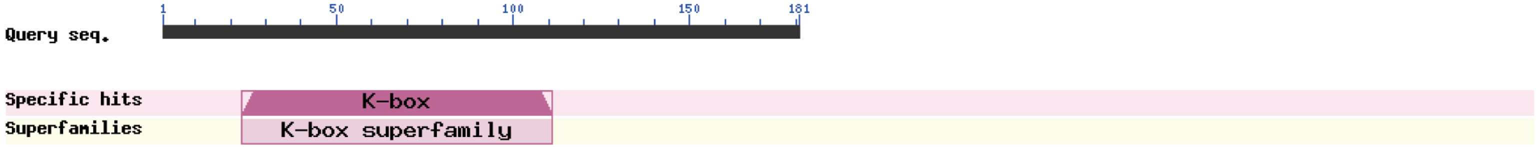


Q42429.1-AP1 similar

**Protein classification:** MADS-box transcription factor is a key regulator of developmental processes, such as meristem identity, flowering time, and fruit and seed development

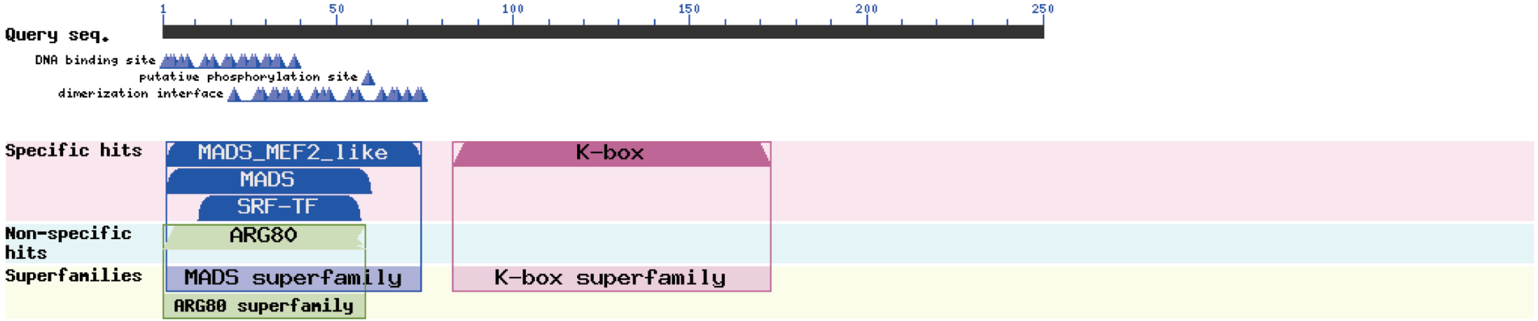

Supplement: Supplementary file 5 [file DataSheet_5.pdf]
