# Supplementary material for: Effects of low temperature on flowering and the expression of related genes in Loropetalum chinense var. rubrum
Source: Front Plant Sci. 2022 Nov 15;13:1000160. doi: 10.3389/fpls.2022.1000160 (PMC9705732; doi:10.3389/fpls.2022.1000160)

augustus63326.t1-FT326

Protein classification: YbhB/YbcL family Raf kinase inhibitor-like protein similar to Arabidopsis thaliana protein FLOWERING LOCUS T and protein TWIN SISTER of FT

CATH: 3.90.280.10 **SCOP:** 4002457

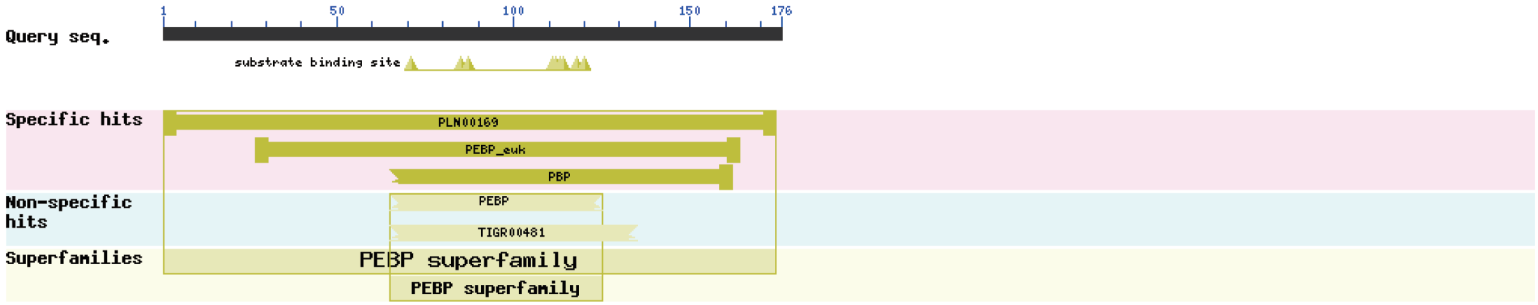

Q9SXZ2.2-F

Protein c

of FT

CATH: 3.90.280.10

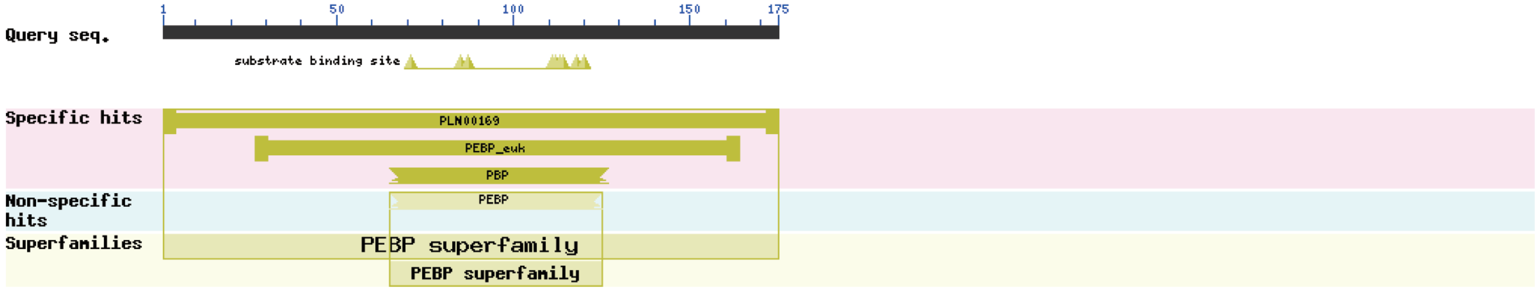

augustus34660.t1-FT660

Protein classification: YbhB/YbcL family Raf kinase inhibitor-like protein similar to Ar complexes with phosphorylated ligands by interfering with kinases and their effectors

CATH: 3.90.280.10 **SCOP:** 4002457

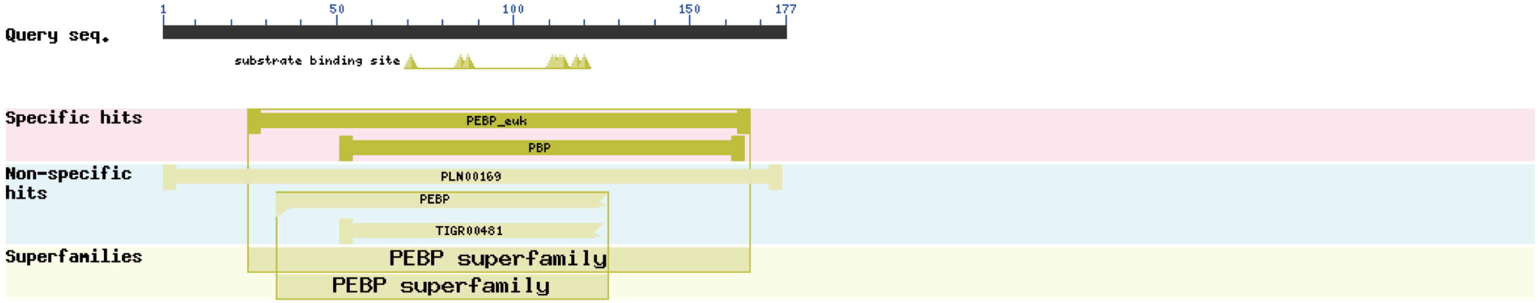

Q9XFK7.1-FT660 similar

tein classification: YbhB/YbcL family Raf kinase inhibitor-like protein similar to Arabidopsis thaliana protein BROTHER of FT and TFL 1 that ma es with phosphorylated ligands by interfering with kinases and their effectors

3.90.280.10 **SCOP:** 4002457

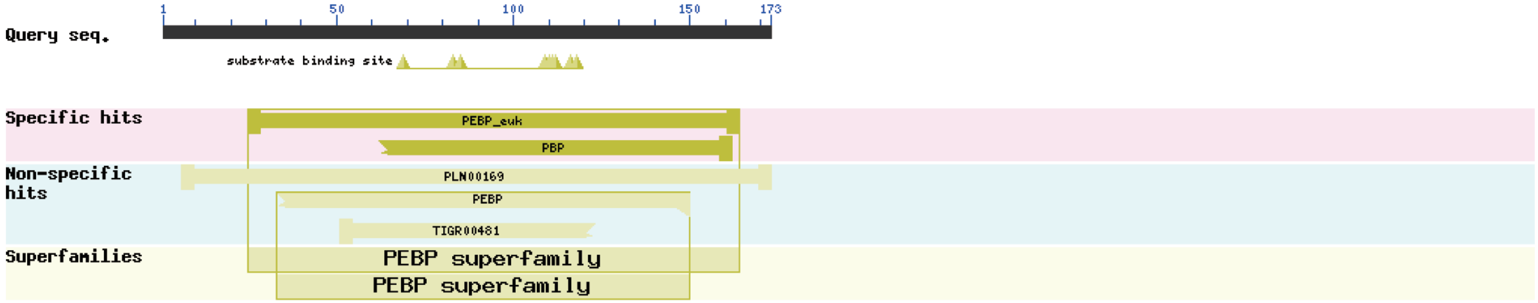

Supplement: Supplementary file 6 [file DataSheet_6.pdf]
