# Supplementary material for: Effects of low temperature on flowering and the expression of related genes in Loropetalum chinense var. rubrum
Source: Front Plant Sci. 2022 Nov 15;13:1000160. doi: 10.3389/fpls.2022.1000160 (PMC9705732; doi:10.3389/fpls.2022.1000160)

augustus27517.t1-FLC

Protein classification: MADS-box transcription factor is a key regulator of developmental processes, such as meristem identity, flowering time, and fruit and seed development

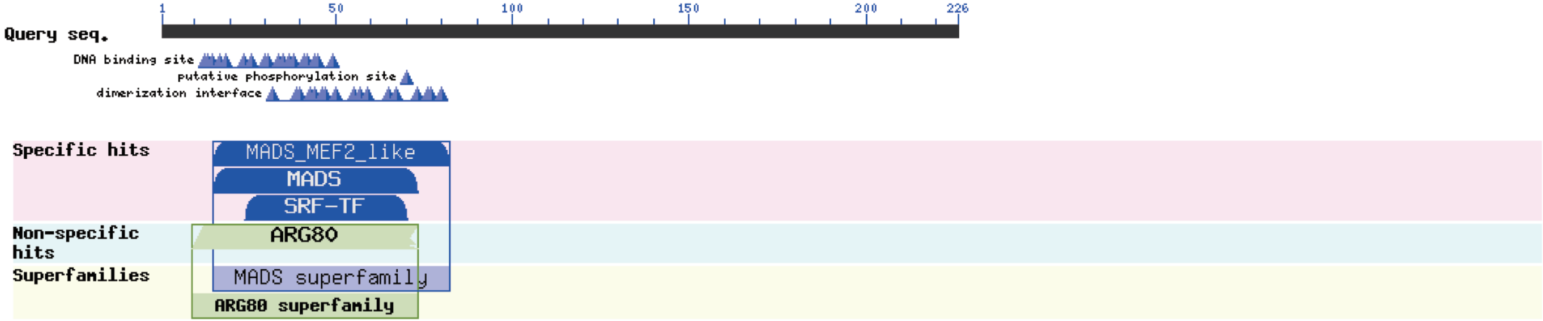

Q9FKK2.1-FLC similar

Protein classification: MADS-box transcription factor is a key regulator of developmental processes, such as meristem identity, flowering time, and fruit and seed development

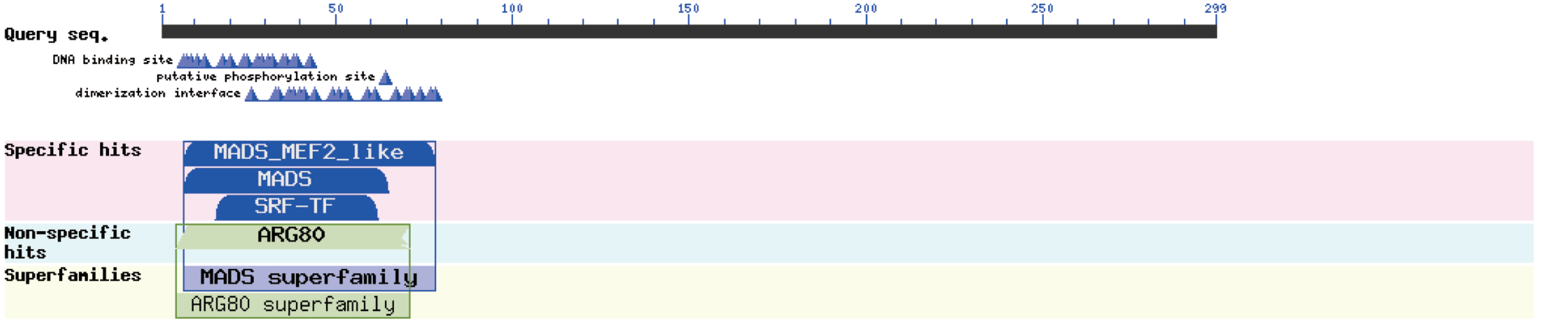

Supplement: Supplementary file 7 [file DataSheet_7.pdf]
