# Supplementary material for: Effects of low temperature on flowering and the expression of related genes in Loropetalum chinense var. rubrum
Source: Front Plant Sci. 2022 Nov 15;13:1000160. doi: 10.3389/fpls.2022.1000160 (PMC9705732; doi:10.3389/fpls.2022.1000160)

augustus15221.t1-SVP

Protein classification: D-hexose-6-phosphate mutarotase catalyzes the interconversion of hexose alpha and beta anomers

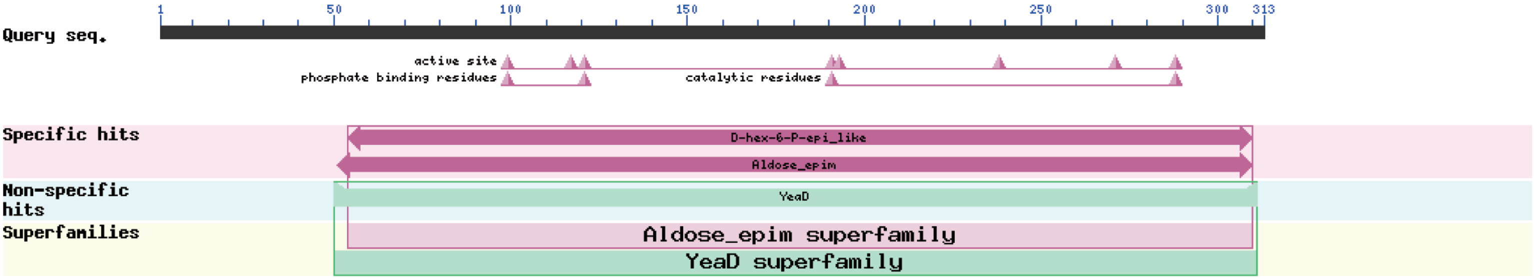

Q40784.1-SVP similar

Protein classification: D-hexose-6-phosphate mutarotase catalyzes the interconversion of hexose alpha and beta anomers

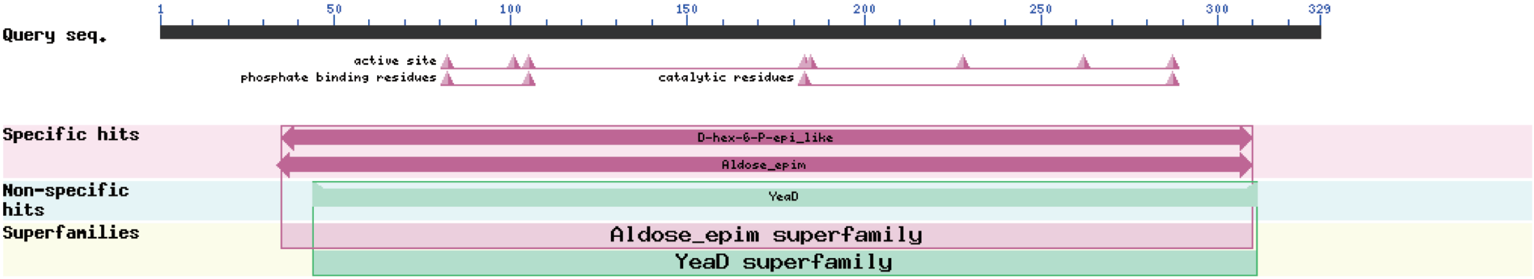

Supplement: Supplementary file 8 [file DataSheet_8.pdf]
