# Supplementary material for: Effects of low temperature on flowering and the expression of related genes in Loropetalum chinense var. rubrum
Source: Front Plant Sci. 2022 Nov 15;13:1000160. doi: 10.3389/fpls.2022.1000160 (PMC9705732; doi:10.3389/fpls.2022.1000160)

augustus62587.t1-TFL1

Protein classification: YbhB/YbcL family Raf kinase inhibitor-like protein similar to Arabidopsis thaliana protein BROTHER of FT and TFL 1 that may form complexes with phosphorylated ligands by interfering with kinases and their effectors

CATH: 3.90.280.10 **SCOP:** 4002457

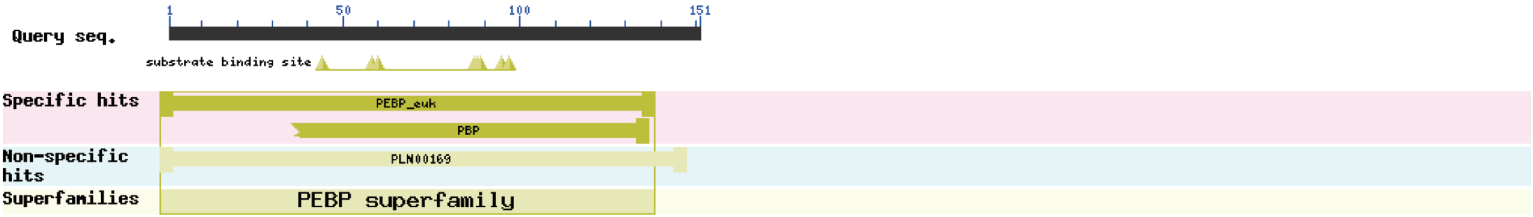

P93003.1-TFL1 similar

Protein classification: YbhB/YbcL family Raf kinase inhibitor-like protein similar to Arabidopsis thaliana protein BROTHER of FT and TFL 1 that may form complexes with phosphorylated ligands by interfering with kinases and their effectors

CATH: 3.90.280.10 **SCOP:** 4002457

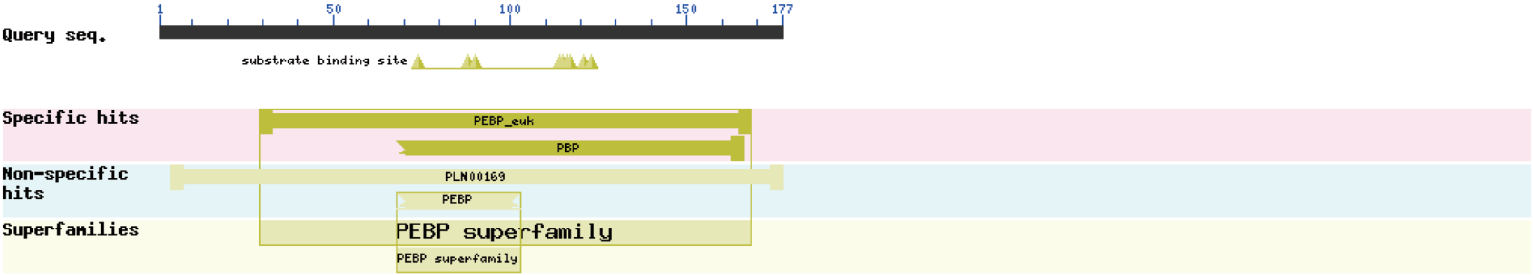

Supplement: Supplementary file 9 [file DataSheet_9.pdf]
